# Association Between First-Trimester Maternal Cytomegalovirus Infection and Stillbirth: A Prospective Cohort Study

**DOI:** 10.3389/fped.2022.803568

**Published:** 2022-03-17

**Authors:** Xinli Song, Qiongxuan Li, Jingyi Diao, Jinqi Li, Yihuan Li, Senmao Zhang, Letao Chen, Jianhui Wei, Jing Shu, Yiping Liu, Mengting Sun, Xiaoqi Sheng, Tingting Wang, Jiabi Qin

**Affiliations:** ^1^Department of Epidemiology and Health Statistics, Xiangya School of Public Health, Central South University, Changsha, China; ^2^National Health Committee (NHC) Key Laboratory of Birth Defect for Research and Prevention, Hunan Provincial Maternal and Child Health Care Hospital, Changsha, China; ^3^Guangdong Cardiovascular Institute, Guangdong Provincial People’s Hospital, Guangdong Academy of Medical Sciences, Guangzhou, China; ^4^Hunan Provincial Key Laboratory of Clinical Epidemiology, Changsha, China

**Keywords:** cytomegalovirus, stillbirth, prospective cohort study, antenatal care, TORCH

## Abstract

**Background:**

Given that the time lag between cytomegalovirus (CMV) screening and diagnosed testing, a better knowledge of the association between pregnant women with CMV screening test positive and stillbirth in an epidemiological perspective was required to assist people being counseled reframe their pregnancy and birth plans based on the magnitude of the risk.

**Methods:**

This study recruited 44048 eligible pregnant women from March 13, 2013 to December 31, 2019. Serological tests including CMV-specific IgM and IgG, and IgG avidity index were used to screen for maternal CMV infection and were measured by automated chemiluminescence immunoassay. The association was assessed using the inverse probability of group-weighted multivariate-adjusted log-binomial models.

**Results:**

A total of 540 infants ended with a stillbirth (12.3 per 1000 pregnancies), and 2472 pregnancies with maternal CMV infection were screened out (56.1 per 1000 pregnancies) among all eligible pregnancies. In the comparison analysis, 326 infants ended with a stillbirth (86.6 per 1000 pregnancies) in the maternal CMV infection group compared with 214 infants (7.8 per 1000 pregnancies) in the group where mothers were not infected with CMV (RR 12.17; 95% CI 9.43–15.71). After excluding the pregnancies of stillbirth with birth defects, a strong association between the two groups was still observed (RR 9.38; 95% CI 6.92–12.70).

**Conclusion:**

Our findings quantified the risk of a woman having a baby with stillbirth if she had a positive serologic CMV screening test in her first trimester, and supported the value of using CMV serologic tests as part of regular testing in pregnant women.

**Trial registration:**

Registered in Chinese Clinical Trial Registry Center; registration number, ChiCTR1800016635; registration date, 06/14/2018 (Retrospectively registered); URL of trial registry record, https://www.chictr.org.cn/showproj.aspx?proj=28300.

## Introduction

Stillbirth was defined as a baby born with no signs of life at 28 weeks’ gestation or more (1). In 2015, an estimated 2.6 million babies (uncertainty range: 2.4–3.0 million) died before birth during the last trimester of pregnancy, meaning a worldwide rate of 18.9 stillbirths per 1000 total births (uncertainty range: 17.4–21.1) (2). Despite improved obstetric and antenatal care, stillbirths have reduced more slowly (Average Annual Rate of Reduction, 1.8%), than either maternal (3.4%) or post-neonatal child mortality (4.5%) since 2000 (2), revealing that stillbirth remained an important public health issue with a large global burden. Although stillbirth had multiple etiologies, there was emerging evidence that viruses cause some stillbirths (3). Human cytomegalovirus (CMV), ubiquitous in nature, was the leading cause of congenital viral infection with an estimated incidence of 0.5–1% of congenital CMV infection in China, and was a known cause of stillbirth (4, 5). It was reported that CMV was detected in fetal tissues and the corresponding placentas from about 16% of stillborn infants, greatly outnumbering other pathogens (6–8), suggesting a strong association between CMV infection in pregnancy and stillbirth. Fetal CMV transmission can occur as a result of either a maternal primary or non-primary infection. The highest rate of congenital CMV infection occurred after primary infections in seronegative mothers (30–40%), while non-primary infections, including CMV reactivations or reinfections, resulted in congenital CMV infection in 0.2–2% of cases, implying that preconceptional immunity may play a role in preventing intrauterine transmission (9).

Maternal CMV screening in early pregnancy proved critical as a secondary preventive strategy in detecting and treating infections in their early stages. In the last decade, major efforts have been made to improve the early laboratory diagnosis of maternal infections (9). Maternal serology was the only reliable screening method in pregnancy that would identify up to 50% of all congenital CMV infections, by performing immunoglobulin M (IgM), immunoglobulin G (IgG), and IgG avidity tests in the population (10, 11). At present, maternal serology was adopted as a regular CMV screening program for pregnant women in China, listed as a crucial item in Chinese Preconception Care Guidelines. If the CMV screening result was positive, a CMV diagnostic test could be performed. The confirming diagnostic test was polymerase chain reaction amplification of CMV-DNA on amniotic fluid that can be collected by amniocentesis from 20 to 22 weeks of gestation or at least 8 weeks after the positive screening test (12, 13). If a woman had a positive serologic CMV screening test in her first trimester, there was a probability of uncovering adverse pregnancy outcomes such as stillbirth and severe fetal anomaly several weeks later, but the probability of this materializing and how it would affect the individual woman who was being counseled remained to be answered. Given that the time lag between screening and diagnosed tests, a better knowledge of the association between pregnant women with CMV screening test positive and stillbirth in an epidemiological perspective was required to assist people being counseled reframe their pregnancy and birth plans based on the magnitude of the risk. Thus, the purpose of this study was to determine the prevalence of pregnant women with a positive serologic CMV screening test in the first trimester, the prevalence of stillbirth, and the risk of women with a positive serologic CMV screening test in their first-trimester pregnancy having a baby with stillbirth. In order to rule out stillbirths caused by factors apart from CMV-mediated impaired placental function, we also explored the association between women who had a positive serologic CMV screening test and stillbirth without those accompanied with major birth defects.

## Materials and Methods

### Data Sources and Study Design

The study was conducted at the Hunan Provincial Maternal and Child Health Care Hospital (Changsha, Hunan Province, China). The present study was a prospective, hospital-based cohort study. The pregnant women, gestation age between gestational week 8 and week 14 (14), admitted to our hospital for prenatal examination from March 13, 2013 to December 31, 2019 were recruited consecutively, and the follow-up was completed before December 31, 2020. Gestational age was calculated according to the last menstrual period (15). Blood samples and some corresponding information were collected at the antenatal first visit, and subsequently, the samples were tested for serologic tests. The outcome was diagnosed in the inpatient department or outpatient clinic. Thus, a total of 44,673 pregnant women were recruited, and 44,048 were included in our final analysis. The present study has been registered in Chinese Clinical Trial Registry Center (registration number: ChiCTR1800016635); date of registration: June 14, 2018. Besides, this study was performed in line with the principles of the Declaration of Helsinki. Approval was granted by the Ethics Committee of Xiangya School of Public Health, Central South University (No. XYGW-2018-36). Informed consent was obtained from all subjects involved in the study.

### Outcome Definition

For international comparison WHO used stillbirth to mean the International Classification of Disease 10th revision (ICD 10) definitions of late fetal deaths: fetal deaths ≥ 1000 gms or ≥28 weeks or ≥35 cm (note birth weight was given priority over gestational age) (1). The birth defects were defined as infants diagnosed in the first year of life with major birth defects according to the classification system of the Chinese Surveillance of Congenital Anomalies of subgroups of major congenital anomalies, and the specific ICD 10 codes referred to the previous study (16).

### Exposure and Covariate

In China, pregnant women routinely underwent serologic screening tests for CMV in the first trimester pregnancy including IgM, IgG, and IgG avidity testing. Confirmed diagnosis of maternal CMV infection in pregnancy should be based on seroconversion in pregnancy (*de novo* appearance of CMV-specific IgG in the serum of pregnant women who were previously seronegative). According to the Chinese Guidelines of CMV Infection Screening Procedures in Pregnancy (17), the type of maternal CMV infection was classified as primary infection or non-primary infection (i.e., following reactivation of a previous infection or reinfection with a new strain). Given that CMV screening tests are typically performed in early pregnancy rather than before conception, and that the serologic test was a screening test rather than a diagnostic test, the present study employed presumed maternal CMV infection rather than confirmed maternal CMV infection (18–20). The definition of presumed maternal primary CMV infection was based on the presence of CMV-specific low-avidity IgG and CMV-specific IgM in the first trimester of gestation (19). Furthermore, because IgG appeared later than IgM when primary infection occurred, pregnant women with CMV IgM but no IgG were asked to reexamine CMV-specific IgG 2–3 weeks later, and subsequently those with CMV-specific IgG in the paired samples were also considered primary CMV infection. The definition of presumed maternal non-primary CMV infection was based on the presence of CMV high-avidity IgG and CMV-specific IgM in the first trimester of gestation (9). In the absence of documented recent seroconversion, it was difficult to completely distinguish between primary and non-primary infection as both can be associated with the presence of IgG and IgM antibodies. Therefore, pregnant women were divided into the maternal CMV infection group and the no maternal CMV infection group, and clinical outcome data were collected and evaluated between the two comparative groups.

Specially trained investigators employed a self-designed questionnaire to obtain the corresponding information. The following was a list of the potential confounders, which were selected based on a review of the relevant literature (21–23): fertilization way (*artificial fertilization or natural fertilization*), age at pregnancy onset (<*25, 25–29, 30–34, or*≥*35*), nation (*Han nationality or others*), areas (*urban or rural*), education level (<*9, 9–11, 12–16, or*≥*17*), pre-pregnancy BMI (<*18.5, 18.5–23.9, 24.0–26.9, 27.0–29.9, or* ≥ *30*), infant sex (*male or female*), gestation (*multiple or single*), parity (*multipara or primipara*), history of stillbirth pregnancy (*yes or no*), history of sexually transmitted diseases (*yes or no*), diabetes before conception (*yes or no*), history of drug abuse (*yes or no*), history of congenital malformations in family (*yes or no*), consanguineous marriage (*yes or no*), active smoking occurred since last menstruation (*yes or no*), passive smoking occurred since last menstruation (*yes or no*), drinking occurred since last menstruation (*yes or no*), folate use (*yes or no*), dyeing hair or perming since last menstruation (*yes or no*), decorating housing since last menstruation (*yes or no*). Having a smoking experience since last menstruation during pregnancy was considered as active smoking exposure. Exposure to secondhand smoke since last menstruation for more than 15 min per day, equal to or more than 4 days a week, for three consecutive months, whether at home and in the workplace was considered as passive smoking. Drinking occurred since last menstruation during pregnancy was defined as drinking exposure. We defined folate use as any use of folic acid in 3 months before pregnancy and/or during the first-trimester pregnancy. Following completion of the questionnaire, the investigator double-checked some of the information by consulting their Maternal and Child Health Manual and medical records. In China, each pregnant woman will be provided with a Maternal and Child Health Manual, which would record their basic demographic characteristics, behavioral habits, illness, and the results of various medical examinations during pregnancy.

We used propensity score estimations and matchings to consider a wide range of baseline characteristics and maternal periconceptional risk factors for stillbirth to isolate the association between pregnant women with CMV screening test positive and having a baby with stillbirth (24, 25). The propensity scores were estimated with a generalized boosted regression model and included all covariates listed in Table 1 as predictors. Notably, these covariates included in model were pre-selected based on literature review and were not, and should not be, selected based on a certain *p*-value level.

**TABLE 1:** The covariates used to define propensity of maternal first-trimester serologic screening CMV testing status.*^a^*

	Maternal serologic screening CMV test			Maximum standardization difference between Groups*^d^*	
	Negative	Positive	*p*-value	Before IPW	After IPW
Fertilization way, Artificial fertilization	9564 (23.00%)	380 (15.37%)	<0.001	0.212	0.015
Age at pregnancy onset			<0.001	0.275	0.003
<25	3678 (11.77%)	268 (14.00%)			
25–29	13989 (44.75%)	1099 (57.42%)			
30–34	10376 (33.19%)	392 (20.48%)			
≥35	3219 (10.30%)	155 (8.10%)			
Nation, The Han nationality	39428 (94.83%)	2390 (96.68%)	<0.001	0.103	0.002
Urban or rural areas, Urban	24472 (58.86%)	1446 (58.50)	0.720	0.007	0.017
Education level (years)			0.024	0.047	0.030
<9	6648 (15.99%)	252 (10.19%)			
9–11	21704 (52.20%)	1460 (59.06%)			
12–16	10206 (24.55%)	614 (24.84%)			
≥17	3018 (7.26%)	146 (5.91%)			
Pre-pregnancy BMI*^c^*			<0.001	0.081	0.042
<18.5	5896 (18.86%)	348 (18.18%)			
18.5–23.9	20430 (65.35%)	1302 (68.03%)			
24.0–26.9	3644 (11.66%)	198 (10.34%)			
27.0–29.9	1013 (3.24%)	38 (1.99%)			
≥30	279 (0.89%)	28 (1.46%)			
Infant sex, Male	22610 (54.38%)	1332 (53.88%)	0.629	0.010	0.006
Gestation			<0.001	0.144	0.007
Multiple	23810 (57.50%)	1240 (50.28%)			
Single	17600 (42.50%)	1226 (49.72%)			
Parity			<0.001	0.465	0.003
Multipara	22536 (54.34%)	1832 (74.59%)			
Primipara	18938 (45.66%)	624 (25.41%)			
History of stillbirth pregnancy	6492 (15.61%)	396 (16.02%)	0.594	0.011	0.002
History of sexually transmitted diseases	98 (0.24%)	12 (0.49%)	0.078	0.036	0.016
Diabetes (preexisting)	318 (0.76%)	14 (0.57%)	0.206	0.026	0.011
Active smoking	1236 (2.97%)	60 (2.43%)	0.089	0.035	0.001
Passive smoking	8642 (20.79%)	518 (20.95%)	0.841	0.004	0.006
Drink	1366 (3.29%)	52 (2.10%)	<0.001	0.082	0.001
History of drug abuse	144 (0.35%)	4 (0.16%)	0.032*^b^*	0.046	0.004
Folate	1836 (4.42%)	140 (5.66%)	0.009	0.054	0.027
Dyeing hair or perming	520 (1.25%)	30 (1.21%)	0.870	0.003	0.019
Decorating housing	2148 (5.17%)	132 (5.34%)	0.709	0.008	0.009
History of congenital malformations in family	42 (0.10%)	20 (0.81%)	<0.001	0.079	0.079
Consanguineous marriage	158 (0.38%)	26 (1.05%)	0.001	0.066	0.005

*Abbreviations: CMV, cytomegalovirus; BMI, body mass index; IPW, inverse probability of group-weighted.*

*^a^Data presented as number (percentage) unless otherwise indicated.*

*^b^The Fisher’s exact probability method was used; otherwise, the χ^2^ test was used.*

*^c^Classification according to Chinese standard for obesity BMI.*

*^d^Inverse probability of group-weighted was estimated by the propensity score from generalized boosted regression. If a standardized difference of less than 0.1 was reached after IPW, the covariates were balanced.*

### Serological Detection

The elbow vein blood of participants (3 mL) was collected for serological tests. CMV-specific IgM antibodies, IgG antibodies and anti-CMV IgG avidity index were measured with an automated chemiluminescence immunoassay (LIAISON XL; DiaSorin, Salugia, Italy). Sera were preabsorbed to avoid false-positive IgM reactions due to rheumatoid factors (26). Samples with concentrations of anti-CMV IgM ≥ 22 U/mL and anti-CMV IgG ≥ 14 U/mL were considered as positive, respectively. Samples with anti-CMV IgG avidity index < 0.20, 0.20 ≤ avidity index ≤ 0.30, and avidity index > 0.30 were considered as low-avidity IgG, moderate-avidity IgG, and high-avidity IgG, respectively.

### Statistical Analyses

The distribution of the patient’s baseline characteristics in the study population was presented as a number (proportion) for categorical data and as the mean ± standard deviation for continuous data. Between-group comparisons were done with the Chi-squared tests or Fischer exact tests, as appropriate. The parameters of generalized boosted regression to estimate the propensity scores were summarized in the Supplementary Table 1. The quality of matching was assessed by standardized differences (similar to an effect size, it was defined as the mean difference divided by the common standard deviation). A covariate with a standardized difference of less than 10% between matched groups was considered well balanced. Next, the inverse probability of group-weighted (IPW) study populations was estimated using the calculated propensity scores from generalized boosted regression (27, 28). The association between first-trimester maternal CMV infection and risk of stillbirth was assessed by relative risk (RR) and their corresponding 95% confidence interval (CI), computed with log-binomial models using the IPW-standardized data.

No correction for multiple testing was applied. Statistical analysis was performed using R software, version 3.5.0 (R Foundation for Statistical Computing). All tests were two-tailed and a *p*-value < 0.05 was considered to indicate a statistically significant difference.

## Results

**FIGURE 1:**
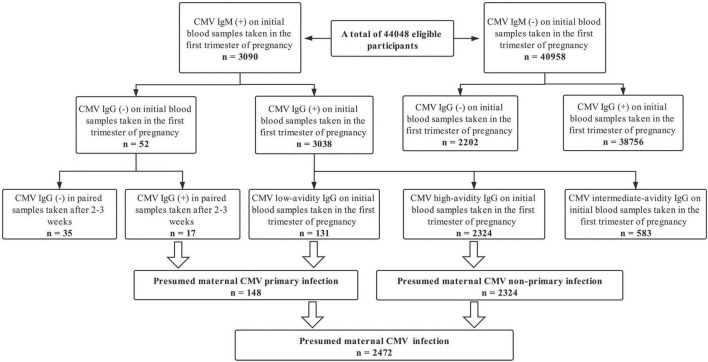
Screening procedure for maternal cytomegalovirus infection during pregnancy among all eligible participants.

The cohort study included 44,048 (98.6%) pregnant women in the primary analysis. A total of 44,673 pregnancies in the first trimester were recruited, 625 (1.4%) excluded: 178 (0.4%) missing CMV-specific serologic tests data; 447 (1.0%) lacking detailed information on pregnancy outcomes. The mean (standard deviation) age at pregnancy onset was 29.4 (4.2) years old. Among all the 44,048 eligible pregnancies, 540 infants ended with stillbirth presenting an incidence rate of 1.23% (range: 1.12–1.33%); 2,472 pregnancies were screened with a positive CMV serologic test presenting a prevalence rate of 5.61% (range: 5.40–5.83%). The screening procedure for maternal CMV infection during pregnancy among all eligible participants was summarized in Figure 1. Among 2472 women with a positive serologic CMV screening test, 94.01% were considered a non-primary infection and 5.99% had a primary infection. In addition, 38756 CMV seropositive pregnant women were screened out, presenting a mean prevalence of CMV-specific IgG of 94.88% (range: 94.68–95.09%) (Figure 1). The distributions of the estimated propensity scores of the comparative groups were shown in Supplementary Figure 1. The propensity scores showed considerable overlap between the comparative groups in these spreads, which indicated excellent covariate balance can be achieved with weights. The estimated propensity scores of the two comparative groups before and after IPW were presented in Table 1. Before IPW, there were statistically significant differences among the comparative groups across most covariates. After IPW, all the covariates were well balanced with the maximum between group-standardized differences < 10%.

The incidence rates of stillbirth in the maternal CMV infection group and the no maternal CMV infection group were presented in Table 2. In the group where women were screened with positive CMV infection results, there were 214 infants ended with stillbirth, with an incidence rate of 8.66% (range: 7.55–9.77%). A total of 326 infants ended with stillbirth in the group where mothers were screened with negative results, presenting an incidence rate of 0.78% (range: 0.70–0.87%). In the comparative analysis of the two groups, the pregnant women with positive CMV screening test in the first trimester of pregnancy showed an 11.17-fold increase in the incidence of stillbirth in newborns (RR 12.17; 95% CI 9.43–15.71) (Table 2). Furthermore, even after excluding the pregnancies of stillbirth accompanied with birth defects, we still observed a 8.38-fold increase in the incidence of stillbirth in newborns (RR 9.38; 95% CI 6.92–12.70).

**TABLE 2:** The association between maternal first-trimester CMV serologic screening test positive and stillbirth in offspring.

Outcome	Total No.	No. with outcome	Incidence rate (95% CI, %)	Relative risk (95% CI)
**Stillbirth**				
No maternal CMV infection	41576	326	0.78 (0.70–0.87)	1.00
Maternal CMV infection	2472	214	8.66 (7.55–9.77)	12.17 (9.43–15.71)
Stillbirth excluded birth defects				
No maternal CMV infection	41576	249	0.60 (0.52–0.67)	1.00
Maternal CMV infection	2472	141	5.70 (4.79–6.62)	9.38 (6.92–12.70)

*Abbreviations: CMV, cytomegalovirus; CI, confidence interval.*

## Discussion

Stillbirth has multiple etiologies and several potential pathogenic mechanisms that could result in the death of the fetus. CMV infection is relatively common, with a high rate of transplacental transmission (29), and has been associated with placental damage that is known to result in fetal malformation and intrauterine death (30). Maternal serology including IgM, IgG, and IgG avidity tests is the reliable CMV screening program. Several countries including China *de facto* offer serologic CMV screening and counseling to advise pregnant women on preventive strategies and eventual laboratory reevaluation (31). Quantifying the risk of women with a positive serologic CMV screening test in their first-trimester pregnancy having a baby with stillbirth is required to assist people being counseled reframe their pregnancy and birth plans based on the magnitude of the risk.

This study observed that the stillbirth rate was estimated to be 12.3 (11.2–13.3) per 1000 births. The stillbirth rate of China in 2015 was higher than our findings, ranging between 5 and 10 per 1000 births (1), which might be due to the growing incidence of stillbirth or the relatively low-quality report data in some less-developed regions of China. The Every Newborn Action Plan aimed for national stillbirth rates of 12 or fewer stillbirths per 1000 births by 2030, but our cohort data revealed that China has not met this target so far. To achieve this target, China should continue to act to reduce preventable stillbirths and strengthen monitoring of stillbirth rates. CMV seroprevalence increased with age and differed by geographic area and socioeconomic status. We also found a very high CMV seroprevalence of about 94.82% among first-trimester pregnancies, which was basically in line with previous literature of 94–98% among Chinese women of child-bearing age (32, 33). The diagnosis of CMV infection in pregnant women based on clinical signs and symptoms is difficult and unreliable since up to 90% of infected mothers are asymptomatic and signs when present are non-specific (rhinitis, pharyngitis, myalgia, arthralgia, headache, fatigue) (34). The gold standard for diagnosis of primary CMV infection is based on serology with evidence of seroconversion documented by the presence of CMV specific IgG in the serum of a pregnant woman who previously tested IgG negative in a serum sample obtained earlier in pregnancy (20). However, this study did not collect the pregestational serum specimens, so the recommended best practice for diagnosing CMV infection is to do a serology test with measurement of IgM, IgG, and IgG avidity (10, 11, 20).

This study employed serologic testing and showed that 5.61% of pregnant women had a positive CMV screening test. Furthermore, pregnant women who had a positive CMV screening test in their first-trimester pregnancy had an 11.17-fold increase in the incidence of newborn stillbirth as compared to those who had a negative CMV screening test (RR 12.17). Our findings were biologically plausible. Congenital CMV infection can cause fetal injury both directly to the fetus and indirectly through placental dysfunction caused by infection or immune-mediated destruction (35). CMV infected and/or bypassed the placenta before it infected the embryo or fetus and was thought to cause adverse pregnancy outcomes that were associated with placental pathology, including intrauterine growth retardation and stillbirth (34, 36). Because fetal growth restriction was related to more than 50% of stillbirths, it was generally considered that both had similar etiologies and risk factors (37). Once there were not enough CMV-specific IgG antibodies to be generated promptly to protect the tissues and organs from infection, CMV would invade the placentas continuously as the gestation age advancing. CMV was able to infect a large spectrum of cells *in vivo* (34), and it could trigger a constellation of molecular mechanisms that altered placental differentiation (38), leading persistent injury fibrosis in infected placentas (39–41). It was suggested that chronic villitis was more frequently in placentas with CMV detected than in those without CMV (42). Chronic villitis including villous infarction, fibrosis, and avascular villi caused thrombosis in main stem and surface vessels, limiting fetal blood flow (8). As a result, abnormal placental hemodynamics and placental dysfunction might result in reduced oxygen and nutrient transport, eventually leading to stillbirth. Although the in-depth mechanisms were not fully elucidated, several possible mechanisms have been proposed, including impairment of trophoblast progenitor stem cell differentiation and function, impairment of extravillous trophoblast invasiveness, dysregulation of Wnt signaling pathways in cytotrophoblasts, and so on (36). Moreover, stillbirths were mainly caused by severe birth defects and fetal growth restriction (43). To rule out stillbirths caused by factors apart from CMV-mediated impaired placental function, we explored the association between women who had a positive serologic CMV screening test and stillbirth without major birth defects, and observed a robust association (RR 9.38).

The current study quantified the risk of a woman having a baby with stillbirth if she had a positive serologic CMV screening test in her first trimester, which answered an important question during pregnancy counseling. A universal screening program for CMV infection in pregnant women has sparked debate (9, 31). This work did not prove an etiological association between CMV and stillbirth, but supported the value of using serologic tests as part of regular CMV testing in pregnant women. Our findings also emphasized the significance of the continuous monitoring of congenital CMV infection as well as intrauterine growth of the fetus in pregnant women with positive serologic testing in their first-trimester pregnancy. In fact, the 11–14 week’ consultation has become an unmissable one worldwide, and it would represent the most practical compromise if only one sample can be taken. Moreover, valaciclovir, that can be safely used in the early fetal period, decreases vertical transmission by 70% and should be implemented as soon as possible after maternal infection (44). Based on long-term experience, we have reasons to believe that maternal screening provides superior and beneficial results in both the short and long terms (9). The limitations of the study needed to be addressed. First, the determination of CMV DNA in maternal biological fluids such as blood and urine would provide additional information. However, in accordance with Chinese situation, the screening method used in this study was based on the Chinese Guidelines of CMV Infection Screening Procedures in Pregnancy, and it also emphasized the significance of serologic testing as a universal screening program for CMV infection among pregnant women. Second, the causal correlation would be more convincing if the CMV DNA testing was conducted in fetus and the related placentas.

## Conclusion

In conclusion, based on the prospective cohort study, we applied the serologic CMV screening test to quantify the risk of women with a positive serologic test in their first trimester pregnancy having a baby with stillbirth, and observed a strong association. Our findings supported the value of using serologic testing as part of regular CMV testing among pregnant women, and also worked in the actual hygiene consultation. When a positive serologic CMV test occurred in the first trimester, continuous monitoring of fetal growth restriction as well as congenital CMV infection of the fetus was supposed to be performed to identify these adverse conditions as early as possible, and thus women would reframe their pregnancies and birth plans according to the magnitude of the risk. To further develop prenatal diagnostic recommendations and optimize prenatal counseling, additional studies evaluating the association between maternal CMV infection and congenital CMV infection, intrauterine growth retardation, and CMV detected in placental and fetal tissue of stillbirths were required in the future.

## Data Availability Statement

The raw data supporting the conclusions of this article will be made available by the authors, without undue reservation.

## Ethics Statement

The studies involving human participants were reviewed and approved by the Ethics Committee of Xiangya School of Public Health, Central South University (No. XYGW-2018-36). The patients/participants provided their written informed consent to participate in this study.

## Author Contributions

JD, JL, and YhL performed the experiments. SZ analyzed the data and statistical analyses. JS, YpL, and MS contributed reagents, material, and analysis tools. XlS, TW, and JQ wrote the main manuscript text. LC, JW, and MS collected reference and managed data. All authors contributed to the article and approved the submitted version.

## Conflict of Interest

The authors declare that the research was conducted in the absence of any commercial or financial relationships that could be construed as a potential conflict of interest.

## Publisher’s Note

All claims expressed in this article are solely those of the authors and do not necessarily represent those of their affiliated organizations, or those of the publisher, the editors and the reviewers. Any product that may be evaluated in this article, or claim that may be made by its manufacturer, is not guaranteed or endorsed by the publisher.
