# Supplementary material for: Association Between First-Trimester Maternal Cytomegalovirus Infection and Stillbirth: A Prospective Cohort Study
Source: Front Pediatr. 2022 Mar 17;10:803568. doi: 10.3389/fped.2022.803568 (PMC8970618; doi:10.3389/fped.2022.803568)
Supplement: Supplementary file 1 [file Data_Sheet_1.PDF]

Supplementary Material

Supplementary Table 1. Summary of parameters of generalized boosted regression to estimate the propensity scores

Parameters	Values
n.trees	20000
interaction.depth	2
shrinkage	0.01
perm.test.iters	0
stop.method	es.mean
estimand	ATT
verbose	FALSE

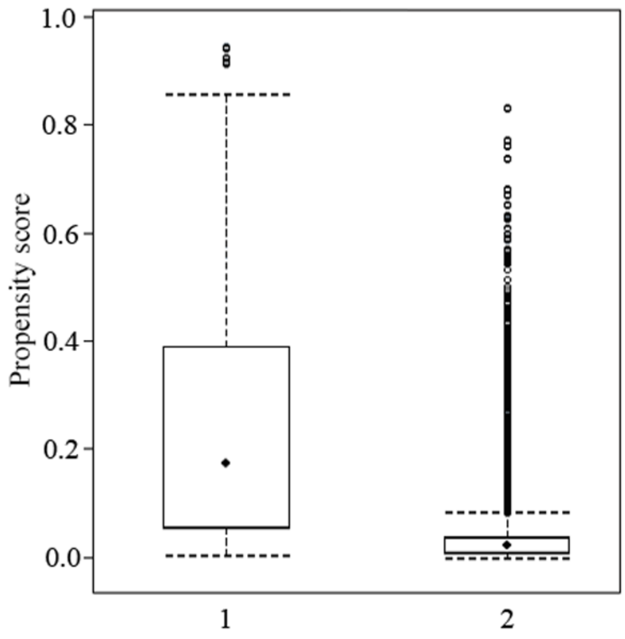

Supplementary Figure 1: The distribution of propensity scores of the two comparative groups

1: No maternal CMV infection group; 2: Maternal CMV infection group.
